# Increased liver stiffness denotes hepatic dysfunction and mortality risk in critically ill non-cirrhotic patients at a medical ICU

**DOI:** 10.1186/cc10543

**Published:** 2011-11-14

**Authors:** Alexander Koch, Andreas Horn, Hanna Dückers, Eray Yagmur, Edouard Sanson, Jan Bruensing, Lukas Buendgens, Sebastian Voigt, Christian Trautwein, Frank Tacke

**Affiliations:** 1 Department of Medicine III, RWTH-University Hospital Aachen, Pauwelsstrasse 30, 52074 Aachen, Germany; 2 MVZ Medical Laboratory Center Dr. Stein and Partner, Wallstrasse 10, 41061 Mönchengladbach, Germany

## Abstract

**Introduction:**

Hepatic dysfunction is a common finding in critically ill patients on the ICU and directly influences survival. Liver stiffness can be measured by the novel method of transient elastography (fibroscan) and is closely associated with hepatic fibrosis in patients with chronic liver disease, but also is increased in patients with acute hepatitis, acute liver failure and cholestasis. We investigated liver stiffness as a potentially useful tool for early detection of patients with hepatic deterioration and risk stratification with respect to short- and long-term mortality.

**Methods:**

We prospectively evaluated 108 consecutive critically ill patients at our medical intensive care unit (ICU) with subsequent longitudinal liver stiffness measurements (admission, Day 3, Day 7 and weekly thereafter) during the course of ICU treatment. Outcome was followed after discharge (median observation time 237 days).

**Results:**

Liver stiffness could be reliably measured in 71% of ICU patients at admission (65% at Day 3, 63% at Day 7). Critically ill patients (*n *= 108) had significantly increased liver stiffness compared to sex- and age-matched standard care patients (*n *= 25). ICU patients with decompensated cirrhosis showed highest liver stiffness, whereas other critical diseases (for example, sepsis) and comorbidities (for example, diabetes, obesity) did not impact stiffness values. At admission to the ICU, liver stiffness is closely related to hepatic damage (liver synthesis, cholestasis, fibrosis markers). During the course of ICU treatment, fluid overload (renal failure, volume therapy) and increased central venous pressure (mechanical ventilation, heart failure) were major factors determining liver stiffness. Liver stiffness values > 18 kilopascal (kPa) at ICU admission were associated with increased ICU and long-term mortality, even in non-cirrhotic patients.

**Conclusions:**

Considering that liver stiffness cannot be validly measured in about 30% of ICU patients, transient elastography performed at ICU admission might be a useful tool to early identify liver dysfunction and predict mortality in critically ill patients at a medical ICU.

## Introduction

Transient elastography of the liver as evaluated by using the FibroScan^® ^(Echosens, Paris, France) instrument is a non-invasive, fast and reproducible tool to assess hepatic stiffness [1,2]. Liver stiffness is calculated from the propagation of an elastic shear wave induced by low amplitude and frequency vibrations that is followed by pulse-echo ultrasonographic acquisitions to measure the velocity of the shear wave propagation. In principal, fast shear wave propagation indicates increased liver stiffness [1]. A strong association of liver stiffness and the degree of liver fibrosis has been demonstrated in various patients with chronic liver diseases, mainly with chronic hepatitis C and non-alcoholic fatty liver diseases [1,3-5]. Its advantage compared to liver biopsy is its fast applicability and non-invasiveness [6]. Importantly, besides FibroScan^® ^(EchoSens), other elastography techniques exist as well, including acoustic radiation force impulse (ARFI, Siemens, Erlangen, Germany), which combines conventional ultrasound and local liver stiffness, as well as similar approaches by supersonic shear imaging (SSI), real-time elastography (RT-E, Hitachi, Wiesbaden, Germany) and shearwave dispersion ultrasound vibrometry (SDUV), or, alternatively, Magnetic Resonance Elastography (MRE), which is based on the MRI detection of acoustic shear waves progressing through the liver tissue [7-11]. Most of the studies evaluating transient elastography focussed on patients with chronic liver diseases in a stable, standard care setting, except for a very few studies performed in patients with either acute hepatic diseases (acute hepatitis, acute liver failure) or acute decompensation of chronic liver disease [12,13]. At present, no systematic examination exists for critically ill patients without a distinct hepatic disease.

In critically ill patients with multiple organ failure, liver dysfunction is very common, as reflected, for instance, by an incidence of 30 to 40% for jaundice in the intensive care unit (ICU) [14,15]. Importantly, liver function is clearly linked to survival in the critically ill [16], as it has been included in most of the widely used clinical multifactorial scoring systems for risk stratification in the ICU setting, for example, Acute Physiology and Chronic Health Evaluation (APACHE-II, cirrhosis as a factor) or Sequential Organ Failure Assessment (SOFA, bilirubin and international normalized ratio (INR) as components) [17]. However, liver dysfunction in the critically ill may be underrated, being more subtle in its clinical presentation and less immediately life-threatening than respiratory, cardiovascular or renal failure [15]. Sophisticated measures such as indocyanine green (ICG) plasma disappearance rate that reflect liver perfusion and function in the critically ill are only available in a few specialized centers [18].

We hypothesized that transient elastography could be a useful tool for the early identification of hepatic dysfunction in critically ill patients without underlying known liver disease and for appropriate risk stratification with respect to ICU and long-term mortality.

## Materials and methods

### Patients and controls

The study protocol conformed to the ethical guidelines of the 1975 Declaration of Helsinki as reflected in *a priori *approval by the local ethics committee. Written informed consent was obtained from the patient, his or her spouse or the appointed legal guardian. We examined 108 patients who were admitted consecutively to the medical intensive care unit at RWTH-University Hospital Aachen, Germany, due to critical medical diseases (Table 1). Patients who were expected to have a short-term (<72 h) intensive care treatment due to post-interventional observation or acute intoxications were not included in the study [19]. Decompensated liver cirrhosis was a clinical diagnosis established by two independent experienced hepatologists based on the patient's history (for example, history of cirrhosis or typical cirrhosis-related comorbidities, such as esophageal varices, variceal bleeding, ascites, hepatic encephalopathy or hepatocellular carcinoma), laboratory values and hepatic imaging (ultrasound, CT, MRI) [20]. Sepsis was defined according to the criteria proposed by the American College of Chest Physicians and the Society of Critical Care Medicine Consensus Conference Committee for severe sepsis and septic shock [19]. None of the patients showed signs of intra-abdominal hypertension or underwent abdominal surgery. The clinical course of patients was observed in a follow-up period (median observation time 237 days, interquartile range 162 to 350 days) by directly contacting the patients, the patients' relatives or their primary care physician [21].

**Table 1:** Baseline patient characteristics and liver stiffness measurements

Parameter	All patients	Sepsis	Non-sepsis
Number	108	80	28
Sex (male/female)	64/44	50/30	14/14
Age median (range) (years)	68 (21 to 90)	68 (21 to 90)	61 (35 to 85)
APACHE-II score median (IQR)	22 (15 to 27)	23 (17 to 28)	19 (7 to 22)
SOFA score median (IQR)	11 (6 to 13)	11 (8 to 14)	9 (1 to 11)
ICU days median (IQR)	9.5 (4 to 26)	13.0 (5 to 28)	7.0 (3 to 16)
Death during ICU n (%)	23 (21%)	20 (25%)	3 (11%)
Death during follow-up n (%)	48 (44%)	41 (51%)	7 (25%)
Mechanical ventilation n (%)	83 (77%)	64 (80%)	19 (68%)
Ventilation time median (IQR) (h)	188 (1 to 1846)	229 (48 to 492)	99 (2 to 312)
pre-existing liver cirrhosis n (%)	8 (7%)	3 (4%)	5 (18%)
ascites (US*) moderate/massive n (%)	11 (10%)/ 6 (6%)	4 (5%)/ 3 (4%)	7 (25%)/ 4 (5%)
pre-existing diabetes n (%)	32 (30%)	21 (26%)	11 (39%)
BMI median (range) (m^2^/kg)	26.0 (22.7 to 29.1)	25.1 (22.5 to 28.2)	26.5 (24.3 to 32.5)
liver stiffness Day 1 median (IQR) (kPa)	9.7 (7.0 to 17.1)	11.0 (7.5 to 18.1)	9.4 (6.3 to 12.2)
liver stiffness Day 3 median (IQR) (kPa)	9.4 (7.3 to 17.7)	10.0 (7.4 to 18.2)	8.4 (6.8 to 27.2)
liver stiffness Day 7 median (IQR) (kPa)	10.1 (6.3 to 18.8)	8.7 (6.1 to 17.4)	11.9 (7.9 to 41.7)

For quantitative variables, median and interquartile range (IQR) (in parenthesis) are given. Abbreviations are: APACHE, Acute Physiology and Chronic Health Evaluation; BMI, body mass index; ICU, intensive care unit; SOFA, sequential organ failure assessment; *ascites detected by abdominal ultrasound (US).

As a control population, 25 sex- and age-matched patients from our standard care unit with various medical diseases, mostly pneumonia or cardiovascular disorders, were examined. Control patients had no evidence of chronic liver diseases (normal liver enzymes, negative serology for viral hepatitis, and regular liver appearance in abdominal ultrasound).

### Routine and experimental laboratory parameters

Patient data, clinical information, blood samples and liver stiffness measurements were collected prospectively. Patients were physically examined daily. Presence of ascites was defined as: (1) none (by ultrasound), (2) moderate (only detectable by ultrasound), (3) massive (already apparent on clinical examination). Furthermore, the presence of peripheral edema (pitting > 2 mm at the lower leg) was assessed, but not quantified. The following experimental parameters were determined from stored serum/plasma samples of all patients and three individual time-points (admission, Day 3, Day 7) according to manufacturers' instructions: coagulation factor VII (STA compact, Roche, Mannheim, Germany, as a marker of liver synthesis function), hyaluronic acid (latex agglutination method, Wako Diagnostics, Osaka, Japan, adapted on cobas e601, Roche; as a serological marker for hepatic fibrosis) [22], cystatin C (BN II, Siemens; as a marker of renal function), NT-proBNP (Modular analytics e170, Roche; as a marker of cardiac function), TNF-alpha (Immulite, Siemens; as a marker of systemic inflammation), and procalcitonin (cobas e411, Roche; as a marker of bacterial infections) [20].

### Liver stiffness measurements

Liver stiffness measurement was performed by transient elastography using the FibroScan^® ^instrument (EchoSens), which employs the propagation of an elastic shear wave delivered by a vibrating probe [6]. This shear wave is followed by an ultrasound wave, and their velocity is measured to determine the elasticity of the liver, expressed in kilopascal (kPa). Per patient, two median liver stiffness measurements at two positions, each consisting of at least 10 individual, valid measurements with a success rate ≥ 60%, were assessed per time-point. If the interquartile range of the valid measurements exceeded 25%, variance was regarded as too high, and values were not deemed reliable. For subsequent statistical analyses, the median of both (median) liver stiffness measurements was employed. All measurements were performed with the 'M probe' (EchoSens). In order to minimize confounding effects due to operator variability, all measurements were performed by one specifically trained, experienced investigator, who was not involved in patient management and blinded to disease etiology. Liver stiffness was assessed at the admission day (within 16 hours after admission to the ICU) and subsequently at Days 3 and 7 during ICU treatment. Consecutively, liver stiffness was then measured weekly in participating patients.

### Statistical analysis

Data are given as median and interquartile ranges (25^th ^to 75^th ^percentile) due to the skewed distribution of most parameters. Differences between two groups were assessed by Mann-Whitney-*U*-test and multiple comparisons between more than two groups by Kruskal-Wallis-ANOVA and Mann-Whitney-*U*-test for *post hoc *analysis [19]. Box plot graphics illustrate comparisons between subgroups, displaying a statistical summary of median, quartiles, range and extreme values. The whiskers extend from the minimum to the maximum value excluding outside (indicated by open circles) and far out values (asterisks), which are displayed as separate points [23]. Correlations between variables have been analyzed using the Spearman correlation tests, where values of *P *< 0.05 were considered statistically significant [24]. Kaplan-Meier curves were plotted to display the impact of liver stiffness measurements on survival. Comparisons of Kaplan-Meier analyses were performed by log rank tests [25]. Optimal cut-off prognostic values for liver stiffness were determined by comparing various log rank tests, testing stiffness values from 12 to 22 [26]. All statistical analyses were performed with SPSS version 12 (SPSS Inc., Chicago, IL, USA)

## Results

### Validity of liver stiffness measurements in critically ill medical patients

**Figure 1:**
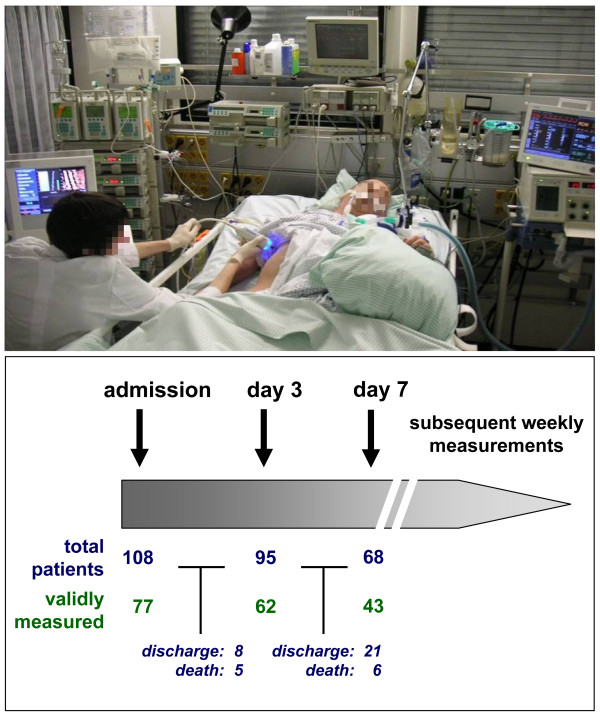
**Liver stiffness measurements in the intensive care unit (ICU) setting**. Critically ill medical patients were included into this study upon admission to the ICU. Transient liver elastography was assessed at admission, on Day 3, on Day 7, and subsequently once per week. The setting of our study is displayed in the upper panel; stiffness measurements were performed at the bedside with a portable FibroScan^® ^apparatus, alongside classical hemodynamic and respiratory monitoring as well as typical ICU treatment measures. The numbers of patients included into the study and the numbers of ICU patients with valid liver stiffness measurements are given in the lower panel of the figure. The numbers of drop-outs, due to transferral to standard care or death, are also displayed.

Liver stiffness is believed to comprise a sum result of hepatic edema, cholestasis and organ fibrosis [12]. However, large studies revealed that liver stiffness measurements fail in about one-fifth of hepatologic patients due to technical reasons in a standard care setting [6]. We examined critically ill patients at a medical ICU in order to evaluate liver stiffness measurements (a) to assess liver function in critical illness and its potential clinical impact, (b) possible prognostic power to predict mortality, and (c) practicability and limitations of transient elastography in intensive care medicine. Therefore, we integrated liver stiffness measurements in the clinical evaluation of critically ill medical patients by assessing stiffness at the admission day, Day 3, Day 7 and, subsequently, weekly during the ICU course (Figure 1). A total of 108 patients were included in our study at admission, with subsequently lower patient numbers at follow-up measurements due to deaths or transfers from the ICU to standard care (Figure 1).

In principal, liver stiffness could be validly measured in 71% of the patients at admission day to the ICU (Figure 1). However, during the course of ICU treatment, involving aggressive fluid challenge, vasopressors, invasive mechanical ventilation and further ongoing severe organ function deterioration [26], the rate of successful liver stiffness measurements decreased to 65% at Day 3 and to 63% at Day 7, respectively (Figure 1). Patients with invalid liver stiffness measurements revealed characteristic features, such as higher body mass index (BMI), obesity (that is, BMI > 25 kg/m^2^) and peripheral edema (Table 2). Measurability was not affected by the disease etiology leading to ICU admission nor by patient's sex. In line with findings reported from standard care patients [6], the rate of invalid stiffness measurements was higher in older ICU patients (Table 2).

**Table 2:** Validity of liver stiffness measurements in critically ill patients*.

	Liver stiffness measurements		*P*-value
	valid	not valid	
**ICU admission **n (%)	77 (71%)	31 (29%)	
sex (male/female, %)	64%/36%	48%/52%	*n.s*.
age median (range) years	64 (21 to 90)	72 (39 to 89)	*0.037*
body mass index median (range) kg/m^2^	24.5 (15.9 to 52.2)	31.2 (22.2 to 66.7)	*< 0.001*
body mass index > 25 kg/m^2^	39%	87%	*< 0.001*
diabetes (%)	31%	34%	*n.s*.
sepsis (%)	75%	71%	*n.s*.
ICU survival/death (%)	79%/21%	77%/23%	*n.s*.
peripheral edema (%)	30%	58%	*0.002*
**ICU Day 3 **n (%)	62 (65%)	33 (35%)	
sex (male/female, %)	68%/32%	48%/52%	*n.s*.
age (median years)	65 (21 to 90)	71 (39 to 89)	*n.s*.
body mass index (median kg/m^2^)	24.0 (15.9 to 52.2)	31.2 (22.5 to 66.7)	*< 0.001*
body mass index > 25 kg/m^2 ^(%)	32%	89%	*< 0.001*
diabetes (%)	28%	36%	*n.s*.
sepsis (%)	79%	70%	*n.s*.
ICU survival/death (%)	84%/16%	78%/22%	*n.s*.
peripheral edema (%)	25%	63%	*< 0.001*
**ICU Day 7 **n (%)	43 (63%)	25 (37%)	
sex (male/female, %)	65%/35%	56%/44%	*n.s*.
age (median years)	65 (21 to 87)	71 (24 to 67)	*n.s*.
body mass index (median kg/m^2^)	23.6 (18.3 to 29.3)	31.2 (24.0 to 66.7)	*< 0.001*
body mass index > 25 kg/m^2 ^(%)	26%	92%	*< 0.001*
diabetes (%)	21%	55%	*0.008*
sepsis (%)	86%	64%	*0.037*
ICU survival/death (%)	88%/12%	88%/12%	*n.s*.
peripheral edema (%)	29%	61%	*0.022*

*Validity of measurements meant the following: 10 measurements each (technical success rate > 60%) from two different anatomical positions and each interquartile range < 25%. From these 2 × 10 measurements, the median of both median values was obtained. If any of these requirements was not met (for example, only one probe position possible, very low success rate at a distinct position, high variance of obtained values, and so on), the liver stiffness value was not considered valid.

### Liver stiffness is increased in critically ill patients, with highest values in decompensated liver cirrhosis, but independent of sepsis

**Figure 2:**
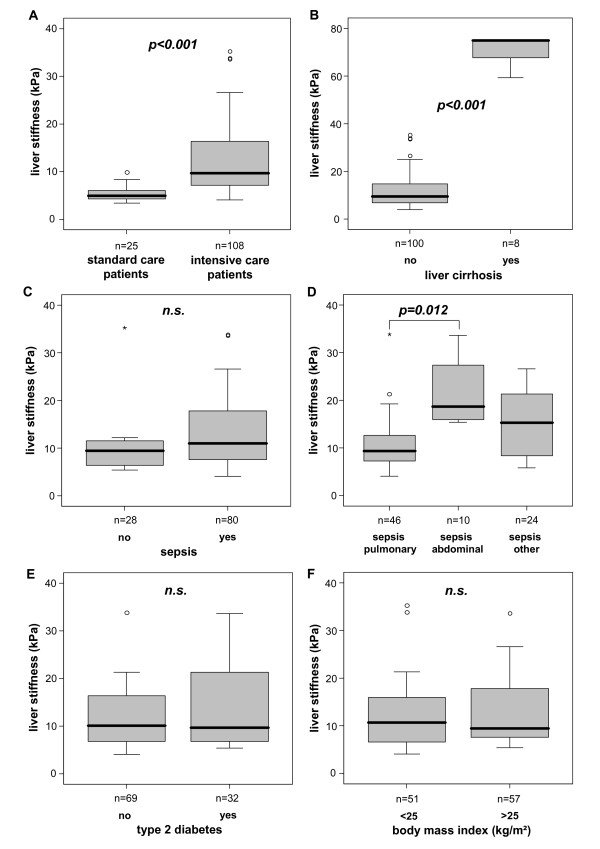
**Liver stiffness in critically ill patients with different disease etiologies at ICU admission**. **(A) **ICU patients have significantly higher liver stiffness values as compared with standard care (control) patients. **(B) **ICU patients with decompensated liver cirrhosis display highest stiffness values. **(C) **Liver stiffness does not differ between sepsis and non-sepsis patients. **(D) **In the subgroup of sepsis patients, patients with an abdominal site of infection show higher liver stiffness than patients with sepsis of pulmonary origin. **(E-F) **Pre-existing type 2 diabetes or obesity (BMI > 25 kg/m^2^) does not affect liver stiffness in ICU patients. *P*-values from U-test are given in the figure.

Based on the validity of liver stiffness measurements in the vast majority of ICU patients, independent of the underlying diagnosis, we next investigated its clinical value in critically ill patients. In comparison to standard care patients without liver disease (as a control cohort), ICU patients had significantly higher liver stiffness values (median 5.0 kPa in controls versus 9.7 kPa in ICU patients, *P *< 0.001, Figure 2A). ICU patients with decompensated liver cirrhosis showed highest liver stiffness values as compared with critically ill patients without cirrhosis (Figure 2B). In decompensated cirrhosis, liver stiffness was found to range from 59 to 75 kPa, thus by far exceeding the average of non-cirrhotic ICU patients (Figure 2B), and confirming prior observations on patients with decompensated liver cirrhosis [1,27].

Cirrhosis and sepsis are both characterized by a hyperdynamic circulation associated with a low systemic vascular resistance and the release of many pro-inflammatory mediators [28]. We, therefore, analyzed whether liver stiffness values can discriminate between septic- and non-septic patients. No difference could be detected between sepsis and non-sepsis patients in liver stiffness at admission (Figure 2C, Table 1); this was also observed when only the non-cirrhotic patients (*n *= 100) were included in the analysis (data not shown). In an extensive subgroup analysis, septic patients with an abdominal site of infection demonstrated higher liver stiffness values in comparison to patients with pulmonary origin of sepsis (Figure 2D). Moreover, in patients with chronic liver diseases, type 2 diabetes or obesity were identified as possible confounders for transient elastography values [3,6]. In our cohort, pre-existing type 2 diabetes or obesity (defined as BMI > 25 kg/m^2^) did not significantly influence liver stiffness, neither in the total cohort (Figure 2E, F) nor for the non-cirrhotic subgroup (data not shown).

### Liver stiffness measurements reflect hepatic function upon admission to the ICU, but non-hepatic organ failure in follow-up examinations during ICU treatment

**Figure 3:**
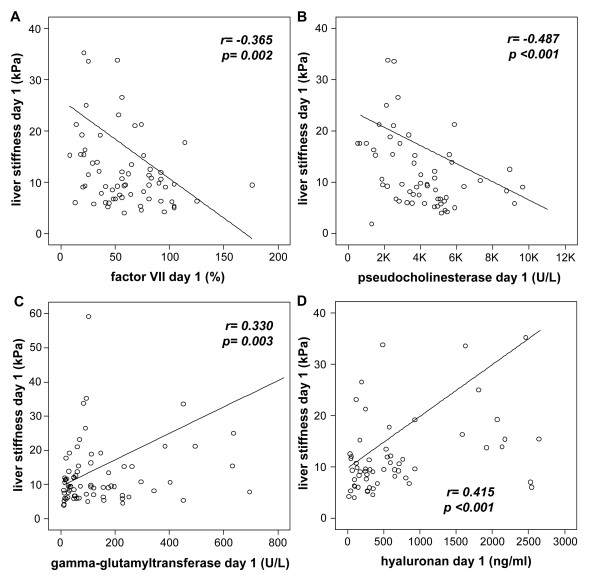
**Liver stiffness in critically ill patients at ICU admission reflects hepatic organ damage**. Liver stiffness values at admission are significantly correlated with biomarkers of hepatic synthesis function (factor VII, **A) **pseudocholinesterase, **B)**, cholestasis (gamma-glutamyltransferase, **C) **and organ fibrosis (hyaluronic acid, **D)**. Correlation coefficient (r) from Spearman rank correlation test and *P*-values are given in the figure.

The marked increase of liver stiffness in all critically ill patients and the maximally elevated values in the subgroup of patients with decompensated cirrhosis, led us to hypothesize that transient elastography examinations at admission to the ICU reflect deterioration of liver function in the critically ill. In fact, liver stiffness at admission was strongly correlated with biomarkers of hepatic synthesis capacity, such as coagulation factor VII activity (Figure 3A), pseudocholinesterase activity (Figure 3B) or international normalized ratio (Table 3). Liver stiffness also correlated with biomarkers of cholestatic damage, namely gamma-GT (Figure 3C), bilirubin and alkaline phosphatase (Table 2). As the critically ill might have pre-existing unrecognized hepatic fibrosis, we also measured hyaluronic acid as a non-invasive biomarker of fibrosis [2,20]. Liver stiffness correlated with hyaluronan serum concentrations in ICU patients (Figure 3D, Table 3).

**Table 3:** Correlations with liver stiffness measurements

			Liver stiffness measurements			
	Admission		Day 3		Day 7	
	r	*P*	r	*P*	r	*P*
**Hepatic organ function**						
***Liver synthesis***						
F VII	-0.365	*0.002*	-0.282	*0.034*	-	*n.s*.
PCHE	-0.487	*< 0.001*	-0.376	*0.009*	-	*n.s*.
INR	0.399	*< 0.001*	-	*n.s*.	-	*n.s*.
AT III	-0.299	*0.012*	-	*n.s*.	-	*n.s*.
***Cholestasis***						
bilirubin	0.489	*< 0.001*	-	*n.s*.	0.415	*0.008*
gamma-GT	0.330	*0.003*	-	*n.s*.	0.343	*0.030*
AP	0.355	*0.005*	-	*n.s*.	-	*n.s*.
***Liver fibrosis***						
hyaluronan	0.415	*0.001*	-	*n.s*.	-	*n.s*.
**Non-hepatic** **organ function**						
***Kidney***						
cystatin C	-	*n.s*.	0.273	*0.044*	0.367	*0.030*
cystatin C GFR	-	*n.s*.	-0.276	*0.042*	-0.377	*0.026*
***Lung***						
ventilation time	-	*n.s*.	0.332	*0.011*	0.573	*< 0.001*
PiP	0.423	*0.001*	-	*n.s*.	-	*n.s*.
PEEP	0.413	*0.001*	-	*n.s*.	-	*n.s*.
***Circulation***						
noradrenalin dose	0.286	*0.013*	-	*n.s*.	0.349	*0.037*
NT-proBNP	-	*n.s*.	0.284	*0.032*	0.478	*0.002*
CVP	-	*n.s*.	-	*n.s*.	0.553	*0.003*
net fluid balance	-	*n.s*.	-	*n.s*.	0.332	*0.045*
**Overall disease severity**						
APACHE II score	0.350	*0.007*	0.304	*0.040*	0.413	*0.023*
SOFA score	0.324	*0.016*	-	*n.s*.	0.562	*0.003*

r, correlation coefficient; *P*, *P*-value (r and *P*-values by Spearman rank correlation); only significant correlations are shown. Abbreviations are: AP, alkaline phosphatase; APACHE, Acute Physiology and Chronic Health Evaluation; AT III, antithrombin III; CRP, C-reactive protein; CVP, central venous pressure; F VII, factor VII; gamma-GT, gamma-glutamyl-transferase; GFR, glomerular filtration rate; ICU, intensive care unit; INR, international normalized ratio; PiP, positive inspiratory pressure; PCHE, pseudocholinesterase; PEEP, positive endexpiratory pressure; SOFA, sequential organ failure assessment

**Figure 4:**
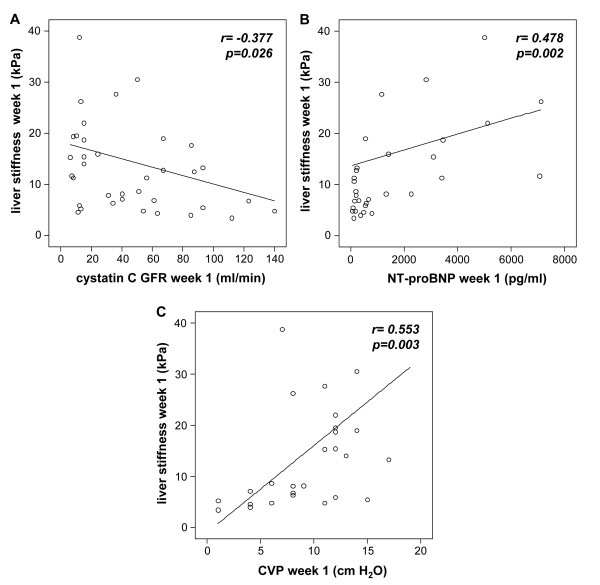
**Liver stiffness after one week of ICU treatment is associated with non-hepatic organ damage**. Liver stiffness values at Week 1 are significantly correlated with biomarkers of renal function (cystatin C-calculated glomerular filtration rate, **A)**, heart failure (NT-proBNP, **B) **and central venous pressure (CVP, **C**). Correlation coefficient (r) from Spearman rank test and *P*-values are given in the figure.

Unexpectedly, most of these associations between hepatic organ function and liver stiffness could not be reproduced anymore at the follow-up measurements at Days 3 and 7 during the course of intensive care treatment. Surprisingly, liver stiffness was then associated with non-hepatic organ functions, specifically with renal failure (Figure 4A, Table 3), pulmonary dysfunction as mirrored by parameters of mechanical ventilation (Table 3) and with circulatory impairment summarizing heart failure and volume load, by the means of vasopressor demand, net fluid balance, NT-proBNP serum concentrations and central venous pressure (Figure 4B, C, Table 3). The median volume of fluid resuscitation that was administered within 24 hours to the patients of our cohort was 6,207 ml (IQR 4,746 to 8,048) at admission day, 4,494 ml (IQR 3,812 to 5,577) at Day 3 and 3,801 ml (IQR 3,298 to 4,471) at Day 7. At all time-points within the first week of ICU treatment, liver stiffness was significantly correlated with clinical composite scores (that is, APACHE-II, SOFA) commonly used at the ICU to determine disease severity (Table 3).

### Liver stiffness values at admission predict ICU- and overall mortality in critically ill patients

**Figure 5:**
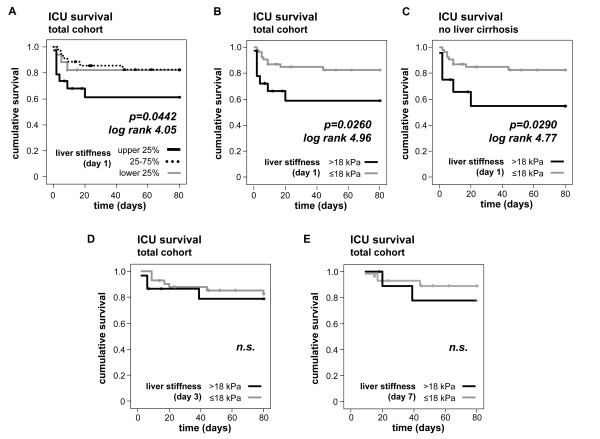
**Prediction of ICU mortality by increased liver stiffness at admission**. **(A) **Kaplan-Meier survival curves of ICU patients are displayed, showing that patients with liver stiffness values of the upper quartile (> 17 kPa, black) at admission have an increased mortality at the ICU as compared to patients with liver stiffness of the lower quartile (< 7 kPa, grey) or of the middle 50% (dotted line). *P*-values and log-rank tests are given in the figure. **(B) **Analysis as in A, using a cut-off liver stiffness of 18 kPa (black line: > 18 kPa, grey line: < 18 kPa). **(C) **Analysis as in B, excluding the ICU patients with decompensated liver cirrhosis. **(D-E) **Liver stiffness measurements at Days 3 or 7 do not predict ICU mortality.

Based on the significant correlations between clinical composite scores and liver stiffness values, we examined whether transient elastography examinations could be useful in predicting ICU- or overall-mortality. Indeed, patients with liver stiffness values of the upper quartile (corresponding to values > 17 kPa) had an increased short-term mortality at the ICU as compared to patients with liver stiffness values of lower quartile or middle 50% (Figure 5A). After testing different possible cut-off values for liver stiffness by Kaplan-Meier curves and log-rank test calculations, a transient elastography value of 18 kPa was found to discriminate best between ICU survivors and ICU non-survivors, when obtained at ICU admission (Figure 5B). In order to rule out potential confounding effects by patients with decompensated liver cirrhosis and extremely high liver stiffness, we confirmed this finding in the critically ill patients after exclusion of decompensated cirrhosis (Figure 5C). However, subsequent liver stiffness measurements at Days 3 and 7 during ICU treatment did not reveal this prognostic relevance to predict ICU mortality (Figure 5D, E). Furthermore, individual changes of liver stiffness ('delta stiffness') within the first week of ICU treatment were not indicative of prognosis either (data not shown).

**Figure 6:**
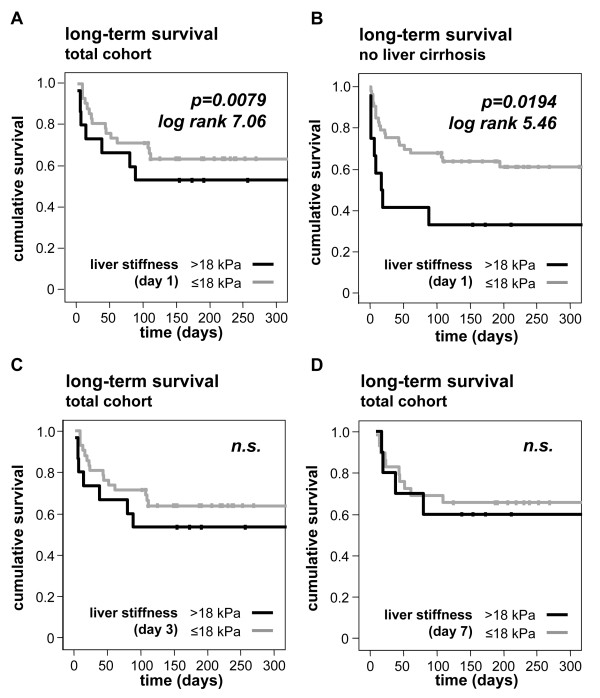
**Prediction of long-term mortality by increased liver stiffness at admission**. **(A) **Kaplan-Meier survival curves of ICU patients are displayed, showing that patients with liver stiffness > 18 kPa (black) at admission have an increased long-term mortality during follow-up as compared to patients with liver stiffness values < 18 kPa (grey). *P*-values and log-rank tests are given in the figure. **(B) **Analysis of A, excluding the ICU patients with decompensated liver cirrhosis. **(C-D) **Liver stiffness measurements at Days 3 or 7 do not predict long-term mortality.

In our cohort, 21% of the critically ill patients died during the course of ICU treatment and an additional 23% died within the first year after discharge from the ICU during the observation period of our study (Table 1). Also, for 'long-term' survival, liver stiffness measurements > 18 kPa upon admission to the ICU predicted mortality in the total patient group (Figure 6A) as well as in non-cirrhotic ICU patients (Figure 6B). Again, liver stiffness obtained in longitudinal measurements at Day 3 or Day 7 of ICU treatment did not predict long-term mortality (Figure 6C, D). Overall, transient liver elastography examinations performed at admission to the ICU appeared capable of identifying patients being at high risk by means of short- and long-term survival.

## Discussion

The measurement of liver stiffness by transient elastography has been recently introduced for the assessment of hepatic fibrosis in patients with chronic liver diseases [1]. So far, it has been mostly validated for viral hepatitis and non-alcoholic fatty liver disease [1-3]. As of yet, no systematic analysis has examined liver stiffness measurements in critically ill patients, in which the liver is often compromised due to different reasons, such as endotoxemia, circulatory alterations (cardiac failure or hyperdynamic state) or exogenous factors (for example, increased intraabdominal or intrathoracic pressure due to impending abdominal compartment or mechanical ventilation, respectively) [28]. Our study revealed that critically ill patients admitted to a medical ICU have significantly elevated liver stiffness compared to standard-care control patients. In line with current literature [29], extremely high values were found in patients with decompensated cirrhosis. Remarkably, the median liver stiffness of non-cirrhotic ICU patients reached values of about 10 kPa, which is commonly regarded as a sign of severe hepatic fibrosis in non-ICU patients [5]. A total of 33% of our medical, non-cirrhotic ICU patients even presented themselves with values > 12.5 kPa at admission, which would otherwise indicate established liver cirrhosis [5].

Smaller studies on selected patient groups have suggested that liver stiffness in patients with acute decompensation might rapidly increase and not reflect hepatic fibrosis *per se*. For instance, patients with acute hepatitis or acute liver failure showed significantly elevated liver stiffness, which decreased after recovery and might be attributed to inflammatory infiltrates in the liver [12,13,30]. Patients with severe cholestasis resulting in increased intrahepatocytic pressure due to impaired bile flow also have elevated liver stiffness values [31]. Vascular factors may also influence liver stiffness measurements, as these values directly correlate with the hepatic venous pressure gradient (HVPG) in patients with portal hypertension [29]. More recently, additional liver-unrelated extrahepatic factors influencing liver stiffness have been identified. By clamping the inferior cava vein in landrace pigs, central venous pressure was found to correlate linearly with liver stiffness [32]. Concordantly, patients with decompensated chronic heart failure also demonstrated high liver stiffness in a small clinical trial [33]. On the other hand, extensive pressure within the liver as reflected by high liver stiffness values might trigger fibrogenic responses in the liver as well, such as activation of hepatic stellate cells and their transdifferentiation into collagen-producing myofibroblasts [34].

In our cohort of critically ill patients from a medical ICU, likely a combination of the above mentioned factors contribute to significantly increased liver stiffness values. However, by simultaneously assessing a wide set of biomarkers and clinical parameters in a large well-characterized patient population, we were able to unravel distinct predominant factors associated with liver stiffness at different time-points in the course of ICU treatment. At admission to the ICU, transient elastography largely reflected hepatic organ dysfunction, as revealed by correlations with biomarkers of liver synthesis, cholestasis and fibrosis. These findings are surprisingly analogous to observations from patients with acute liver failure [12], possibly suggesting that the pathomechanisms affecting hepatic organ function in non-cirrhotic ICU patients are very similar to those in acute liver failure (for example, hepatic edema, reduced hepatic blood flow, cholestasis, extracellular matrix deposition). Therefore, implementing transient elastography examinations into the assessment of critically ill patients at admission could potentially help to early identify affection of the liver related to critical diseases.

The implication of elevated liver stiffness in medical ICU patients substantially changed during the course of intensive care treatment, which is usually characterized by high-volume fluid substitution, vasopressor administration and organ replacement therapies, such as mechanical ventilation and continuous veno-venous hemofiltration [26]. At Days 3 and 7, parameters of non-hepatic organ function, namely kidney, lung and heart/circulation, were correlated with liver stiffness, but liver stiffness was not closely associated with biomarkers of hepatic function anymore. Likely, these associations can be referred to two major pathogenic mechanisms: (a) fluid overload due to therapeutic measures and renal failure, and (b) elevated central venous pressure caused by positive pressure ventilation and impaired cardiac function. The latter mechanism has been proposed by two smaller studies in patients with decompensated heart failure, in which liver stiffness was correlated to reduced left ventricular ejection fraction and pathologically elevated NT-proBNP serum levels [32,33], as also seen in our study.

Besides identifying factors influencing liver stiffness in critical illness, we provided evidence that transient elastography measurements were clinically relevant for predicting mortality in a prospective setting. Patients with liver stiffness values > 18 kPa had significantly reduced survival rates not just during the course of ICU treatment, but also in long-term observation. Apart from our study, data on the prognostic value of transient elastography for predicting 'hard end-points', such as mortality, are very limited. One recent study demonstrated that high liver stiffness indicates poor survival in patients with hepatitis C [35]. In addition, an association of high liver stiffness with development of hepatocellular carcinoma and mortality has been reported in patients with HBeAg-negative hepatitis B [36]. In patients with various chronic liver diseases, transient elastography was found to predict the occurrence of complications related to portal hypertension [29].

### Limitations of the study

Some limitations of our study need to be considered. First, we cannot provide liver histology from our ICU patients, as there has been no clinical necessity to perform liver biopsies in this critically ill patient cohort. In order to overcome this potential short-coming (lack of liver histology), we had included hyaluronic acid as a serological fibrosis marker. Second, data on possible associations between extensive hemodynamic monitoring and liver stiffness are lacking, as echocardiography, pulmonary artery catheterization or Pulse Contour Cardiac Output (PiCCO) recording were only performed in a subgroup of our patients, which did not allow sufficient statistical analysis. Therefore, further studies are warranted focussing on hepatic dysfunction, potentially also as a therapeutic target, in critical illness. Another possibly relevant aspect for clinical applicability of this technique is that the measurements in our cohort have been performed by a single investigator in order to exclude confounding inter-observer effects. However, feasibility of liver stiffness measurements in clinical practice, when performed by multiple less experienced investigators, might be lower than anticipated from our study. Of note, a different probe for obese patients ('XL probe') has been recently released by the distributor of the FibroScan^® ^apparatus, which might possibly help increase the number of ICU patients that could be validly measured by transient elasography. Alternatively, other techniques assessing liver stiffness might be tested in the ICU setting as well, for example, ultrasound-based ARFI detection, because some of these techniques might have a higher success rate, especially in the presence of edema or ascites [7,37]. Moreover, we would like to stress that the potential threshold of 18 kPa for liver stiffness upon ICU admission needs to be prospectively validated in an independent cohort with respect to relevant outcome measures.

## Conclusions

We herein prove for the first time the applicability of transient elastography in a large and well-characterized, prospectively analyzed cohort of critically ill medical patients. Liver stiffness reflects several aspects of hepatic organ function when measured upon ICU admission. During the course of intensive care treatment measures, fluid overload and increased central venous pressure are major factors determining liver stiffness. Considering that liver stiffness cannot be validly measured in about 30% of ICU patients, transient elastography performed at ICU admission might be a useful tool to predict mortality in critically ill, even in non-cirrhotic patients, and further prospective validation studies using the identified cut-off values should be performed. This evident link between mortality and liver stiffness emphasizes that affection of the liver is common in critical disease and is decisive for the patient's individual chance of survival.

## Key messages

• Measuring liver stiffness by transient elastography is a novel tool commonly used by hepatologists to assess hepatic fibrosis in patients with chronic liver diseases, and comprises a sum result of hepatic edema, cholestasis and organ fibrosis

• In a large prospective study measuring liver stiffness repetitively during the course of ICU treatment, critically ill patients without known liver disease had significantly elevated liver stiffness, reaching levels commonly observed in advanced hepatic disease, and showed the highest values in decompensated liver cirrhosis

• At admission to the ICU, liver stiffness is closely related to hepatic damage (liver synthesis, cholestasis, fibrosis), but during the course of ICU treatment, fluid overload (renal failure, volume therapy) and increased central venous pressure (mechanical ventilation, heart failure) are major factors determining liver stiffness

• Liver stiffness values > 18 kPa at ICU admission were associated with increased ICU and long-term mortality (observation period about one year), indicating that transient elastography performed at ICU admission might be a valid tool for the early identification of liver dysfunction and the prediction of mortality in critically ill medical patients

## Abbreviations

APACHE: Acute Physiology and Chronic Health Evaluation; AP: alkaline phosphatase; ARFI: acoustic radiation force impulse; AT III: antithrombin III; BMI: body mass index; CRP: C-reactive protein; CVP: central venous pressure; ELISA: enzyme-linked immunosorbent assay; yGT: gamma-glutamyl-transferase; GFR: glomerular filtration rate; HVPG: hepatic venous pressure gradient; ICG: indocyanine green; ICU: Intensive Care Unit; INR: international normalized ratio; kPa: kilopascal; MRE: Magnetic Resonance Elastography; NT-proBNP: N-terminal pro-brain natriuretic peptide; *P*: *P*-value; PCHE: pseudocholinesterase; PEEP: positive endexpiratory pressure; PiCCO: Pulse Contour Cardiac Output; PiP: positive inspiratory pressure; r: correlation coefficient; SAPS: simplified acute physiology score; SDUV: shearwave dispersion ultrasound vibrometry; SIRS: systemic inflammatory response syndrome; SOFA: sequential organ failure assessment; SSI: supersonic shear imaging.

## Competing interests

The authors declare that they have no competing interests.

## Authors' contributions

AK, AH, CT and FT designed the study, analyzed data and wrote the manuscript. AH performed liver stiffness measurements. EY performed experimental laboratory measurements, and HD, ES, JB, LB and SV collected data and assisted in patient recruitment. All authors read and approved the final manuscript.
